# Assessing the Effectiveness of Dowel Bars in Jointed Plain Concrete Pavements Using Finite Element Modelling

**DOI:** 10.3390/ma18030588

**Published:** 2025-01-28

**Authors:** Saima Yaqoob, Johan Silfwerbrand

**Affiliations:** Division of Concrete Structures, Department of Civil & Architectural Engineering, KTH Royal Institute of Technology, SE-10044 Stockholm, Sweden; jsilfwer@kth.se

**Keywords:** jointed plain concrete pavement, finite element modelling, load transfer efficiency, steel dowel bar, plate dowel bar, flexural stress

## Abstract

Aggregate interlocking and dowel bar systems are the two primary mechanisms in a jointed plain concrete pavement for transferring the wheel loads from the loaded slab to the adjacent unloaded slab, avoiding critical stresses and excessive deformations across the joint. Aggregate interlocking is suitable for small joint openings, while the dowel bar provides effective load transmission for both smaller and wider joint openings. In this study, a three-dimensional finite element model was developed to investigate the structural performance of dowelled jointed plain concrete pavements. The developed model was compared with an analytical solution, i.e., Westergaard’s method. The current study investigated the effectiveness of the dowel bars in jointed plain concrete pavements considering the modulus of elasticity and the thickness of the base layer, as well as dowel bar diameter and length. Furthermore, the load transfer efficiency (LTE) of a rounded dowel bar was compared with that of plate dowel bars (i.e., rectangular and diamond-shaped dowel bars) of a similar cross-sectional area and length. This study showed that the LTE was enhanced by 4% when the base layer’s modulus of elasticity increased from 450 MPa to 6000 MPa, while the increase in stress was 23%. A 1.2% improvement in the LTE and a 2.1% reduction in flexural stress were observed as the base layer’s thickness increased from 100 to 250 mm. Moreover, increasing the dowel bar’s diameter from 20 mm to 38 mm enhanced the LTE by 4.3% and 3.8% for base layer moduli of 450 MPa and 4000 MPa, respectively. The corresponding rise in stresses was 10% and 5%. The diamond-shaped dowel bar of a 50 × 32 mm size showed a 0.48% increase in the LTE, while sizes of 100 × 16 mm and 200 × 8 mm reduced the stress 6.7% and 23.1%, respectively, compared to that in the rounded dowel bar. With rectangular dowel bars, a 4% rise in the stress was noted compared to that with the rounded dowel bar. Increasing the length of the diamond-shaped dowel bar slightly improved the LTE but had no impact on the stress in the concrete slab. The findings from this study can help highway engineers improve pavements’ durability, make cost-effective decisions, contribute to resource savings in large-scale concrete pavement projects, and enhance the overall quality of infrastructure.

## 1. Introduction

Concrete pavements provide efficient and safe surfaces for the movement of heavy vehicles, primarily subjected to traffic and thermal stresses. Jointed plain concrete pavements (JPCPs) are plain cast-in-place concrete pavements constructed with a certain joint spacing. Joints are essential elements in the design of JPCPs that are introduced by saw-cutting in the transverse and longitudinal directions to control the natural cracks which can occur due to shrinkage and curling of the concrete slab. It has been reported that plain concrete pavements, in the absence of joints, can become crammed with cracks within a short time, i.e., within 1–2 years of construction [1]. JPCP does not contain any reinforcement, and the inclusion of joints creates a break in the continuity of the slab and reduces the load transfer between the adjacent slabs, and this inadequate load transfer may generate higher stresses and deflection at the corners or edges, which might result in various types of pavement damage, such as corner cracking, spalling, faulting, and pumping, thus jeopardising the performance of the pavement system [2,3].

In JPCP, the load transfer across the joint is primarily achieved through aggregate interlocking and dowel bar action. Aggregate interlocking is a load transfer mechanism that relies on the rough surface of the adjacent slabs or the interaction between the aggregate particles at the joint. Aggregate interlocking works well for small joint openings and low-volume traffic. However, for wider joints and heavy vehicle loads, dowel bars are more effective in transferring the load from one slab to the abutting slab without restricting the joint movement, caused by the expansion and contraction of the slabs. Furthermore, the dowel bars also help in keeping the slabs aligned [4,5]. The load transfer across the joint in a JPCP can be defined by Equation (1) [6].

(1) $$LTE_{\delta }=\frac{\delta_{u_{L}}}{\delta_{L}}\times 100$$

where

$LTE_{\delta }$ = the load transfer efficiency based on deflection
$\delta_{u_{L}}$ = the deflection of the unloaded slab at the joint
$\delta_{L}$ = the deflection of the loaded slab at the joint

Dowel bars are typically 500–600 mm long and 25–40 mm in diameter and are placed at the mid-depth of the concrete slab, with a spacing of 250–300 mm in the direction of traffic [7]. In the Czech Republic, a standardised size for the dowel bars (500 mm in length and 25 mm in diameter) is used for all pavement thicknesses, whereas in Slovakia, the dowel size is recommended according to the concrete slab’s thickness [8,9,10,11]. Before placing a dowel bar in the dowel slots, which are either cut or drilled into the cast-in-place concrete pavement or fabricated in the jointed precast concrete pavement (a modern technique used to repair damaged concrete pavement) [12,13,14,15,16], the dowel bars are coated with epoxy and primed with lubricant compound to prevent corrosion and avoid bonding with the surrounding grout, respectively. Once they have been prepared, the dowel bar chairs, endcaps, and foam-core board are fitted onto the dowel bar; for more details, see [17,18,19,20]. However, when concrete pavements are laid using slip-form pavers, the machine automatically installs the dowel bars without requiring any additional support.

Several studies have been conducted on optimising the performance of JPCP using both numerical and experimental approaches. Shoukry et al. (2003) studied the stresses in the concrete surrounding a dowel bar and observed that the stresses are lower in the concrete around dowel bars treated with a debonding agent compared to that around uncoated dowel bars [21]. Breemen (1955) conducted experimental tests to study the free movement of the dowel bars in concrete slabs, using dowel bars with different coatings and protective treatments [22]. Nishizawa (2001) found that the stress in a concrete slab is less affected by the spacing between the dowel bars, while the spacing has a significant effect on the stresses in the dowel bars [23]. Moreover, Keymanesh et al. (2018) examined the spacing of dowel bars and found that it substantially affects the dowel bars’ load transfer efficiency [24]. Sadeghi and Hesami (2018) investigated the load transfer efficiency (LTE) of JPCP and found that it can be improved by increasing the thickness of the concrete slab [25].

Kim et al. (2018) studied the effect of the dowel bar arrangements on the stress and deflection in the slabs by comparing standard and special dowel bar configurations. In the standard case, the dowel bars were spaced along the entire slab’s width at a spacing of 305 mm, while in the special arrangement, only three dowel bars were placed along each wheel path. The findings indicated no significant differences in the stress or deflection between the two dowel bar arrangements [26]. Swarna et al. (2024) analysed the impact of wider concrete slabs on the tensile stress and concluded that wider slabs substantially increase the tensile stress in the longitudinal direction while having a slight effect on the transverse stress [27]. Yaqoob et al. (2024) discovered that the LTE is significantly reduced by increasing the joint width between adjacent slabs [28]. Wang et al. (2022) observed that increasing the joint spacing in JPCPs can lead to a higher occurrence of transverse cracking and faulting [29]. Li et al. (2023) conducted both experimental and numerical investigations to examine the LTE of a prefabricated cement concrete pavement considering the presence and absence of transverse tongue and groove joints, slab thickness, and joint rigidity. Their findings suggested that the LTE is slightly lower in a slab with a transverse joint. Furthermore, increasing the joint rigidity enhances the load transfer, while it decreases with increasing slab thickness [30]. Mackiewicz (2015) analysed the impact of the dowel bar diameter and spacing in concrete pavement. He reported that using small diameters can increase the risk of damage within the concrete slab [31].

A significant concern in concrete pavement is that the dowel bars often corrode, reducing their cross-sectional area and building up corrosion products, affecting the dowel bars’ smoothness and leading to greater movement restriction during the concrete slab’s expansion and contraction. As mentioned in Section 1, dowel bars are coated with a thin layer of epoxy to prevent corrosion, but the epoxy layer can easily be worn away under repeated traffic loading. Previous studies have shown that glass-fibre-reinforced polymer (GFPR) dowel bars are corrosion-free, and due to their smoother surface, they reduce the stresses at the dowel–concrete interface. Although GFRP dowel bars have relatively low stiffness compared to that of traditional steel dowel bars, they can achieve an equivalent performance in terms of the LTE, deflection, and concrete bearing stress by requiring reduced spacing or a 20–30% larger diameter [32,33,34,35,36,37].

It has also been reported that the structural response of concrete pavement is greatly affected by the positioning and alignment of the dowel bars (horizontal and vertical skew). In the study by Smith and Benham (1938), the impact of misalignments in the concrete slabs (127 mm and 152 mm thick) was evaluated, and they reported that in 127 mm thick concrete slabs, spalling occurred with 6 mm of misalignment, whereas in 152 mm thick slabs, a 25 mm misalignment was enough to cause spalling [38]. However, Segner and Cobb (1967) found that horizontal alignment had less impact than vertical alignment [39]. Cong and Ling (2016) reported that a 15° deviation in the vertical angle resulted in a 12% reduction in the load transfer efficiency [40]. Peng et al. (2012) conducted experimental tests to evaluate the impact of deviations on bitumen-treated dowel bars and plastic-sprayed dowel bars. They found that regardless of the dowel bar’s surface treatment, the load transfer decreases with an increase in the deviation angle [41]. A study conducted by Leong et al. (2006) concluded that a concrete slab can be cracked if the dowel bar misalignment tolerance exceeds 25 mm [42]. This is because the misalignment of the dowel bars may lock the joint movement during the expansion and contraction of the concrete slabs, which may lead to cracking at the joints [43,44,45].

## 2. Research Significance

In Sweden, very few pavements are constructed as concrete pavements, with asphalt being the prevalent material. Although some pavements have shown a remarkable performance beyond expectations, others have experienced damage earlier than expected. The latest construction occurred in 2006 on a European highway that links Mehdeby and Uppsala [46], reflecting Sweden’s insufficient practical experience in the design, construction, and maintenance of concrete pavements [47,48]. A notable exception is the ongoing Stockholm Bypass (Förbifart) project, a 21 km long tunnel connecting Kungens Kurva in the South to Hägvik in the north. Initially, the tunnel opted for concrete as the initial choice of material, but ultimately, asphalt was selected [47]. The Swedish Transport Administration (Trafikverket) attributed this decision to the potential difficulty of efficiently repairing concrete pavements in the case that any damage occurred. In recent years, increasing traffic volumes and the ability of concrete pavements to sustain heavy vehicle loads without their performance being compromised have prompted the Swedish Transport Administration to rebuild expertise in the field of concrete pavements [49,50].

In Sweden, Yaqoob et al. (2021) reported that rehabilitating concrete pavements using precast concrete slabs has dramatically reduced the repair time. However, the dowel bar is a key factor in ensuring the reliable performance of precast slabs [51]. In this paper, a three-dimensional finite element model was developed using Abaqus software to investigate the effectiveness of dowel bars in terms of the load transfer efficiency and flexural tensile stresses at the bottom of the concrete slabs based on pavement-related parameters, i.e., the Young’s modulus and thickness of the base layer and the dowel bar’s diameter and length. The accuracy of the model was checked by comparing it with the Westergaard method. Furthermore, the pavement behaviour was also evaluated for different shapes of dowel bars (i.e., rectangular- and diamond-shaped dowel bars) based on different cross-sectional sizes. The results revealed that the load transfer through the dowel bar is more efficient when the base layer is not of a high stiffness. Furthermore, the dowel bar’s diameter and the dowel bar having a diamond shape are more influential in enhancing the pavement’s performance. The findings of this study could contribute to resource savings, reduce construction and maintenance costs, and provide long-term social benefits in the field of concrete pavements.

## 3. Finite Element Modelling of Dowelled Jointed Plain Concrete Pavement

A three-dimensional finite element model of jointed plain concrete pavement was developed in the Abaqus program [52]. The model included concrete slabs and three underlying layers i.e., the base, subbase, and subgrade. In Sweden, the typical dimensions of a JPCP slab are 5.0 m × 3.5 m [53]. However, to reduce the computational time, only half of the concrete slab was modelled in this study, resulting in a model with dimensions of 3.0 m in length and 1.75 m in width. This reduction in slab size is justified by the fact that the load is applied near the edge, meaning it will not significantly affect the dowel bars located farther away from the load. The slab had a thickness of 200 mm, while the thickness of the base, subbase, and subgrade layers was 150 mm, 300 mm, and 2500 mm, respectively [54]. A joint opening of 6 mm between the concrete slabs was considered, and the slabs were connected with steel rounded dowel bars. The dowel bars were 32 mm in diameter, 406 mm long, and spaced 300 mm centre to centre at the mid-height of the concrete slab.

**Figure 1:**
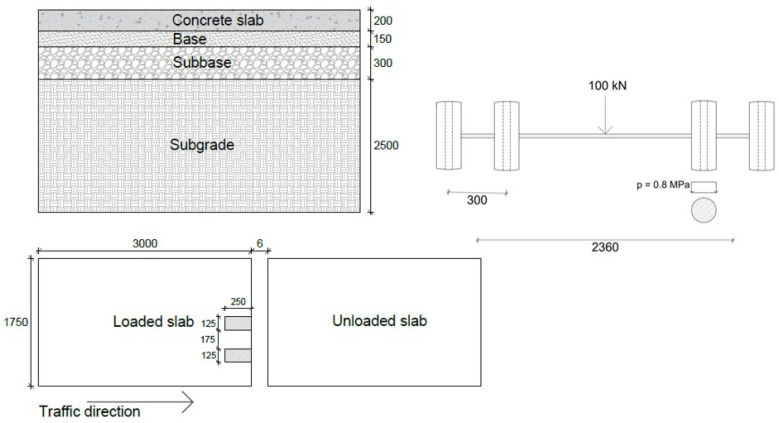
Geometrical and loading configuration of 3D model [all dimensions are in mm].

A vehicle load was simulated using a standard axle configuration, consisting of a single axle with dual wheels on either side. The overall load of the axle is 10 metric tons, and each tire on a standard axle constitutes 25 kN, with a pressure of 800 kPa [55]. Half of the axle was modelled using dual-wheel loads assumed to be static and applied with a uniform distribution over a rectangular plate with 125 mm × 250 mm dimensions, positioned 225 mm from the edge of the loaded slab. The geometrical and loading configurations of the 3D model are shown in Figure 1. The focus of this study was to capture the fundamental interaction within the pavement system; therefore, the concrete slab base, subbase, and subgrade and the steel dowel bar were considered elastic materials, and each material’s properties are presented in Table 1 [56]. However, in the real world, the materials may exhibit some inelastic or viscoelastic behaviour due to environmental factors such as temperature variations, moisture content, or long-term aging and creep. To capture real-world conditions accurately, it is important to consider a complex material model, but this can be a challenging effort.

**Figure 2:**
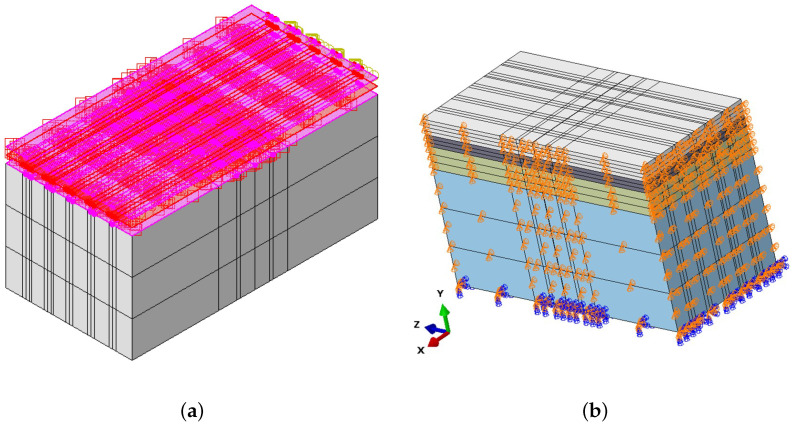
Three-dimensional views of the model: (**a**) interaction and (**b**) boundary conditions.

**Figure 3:**
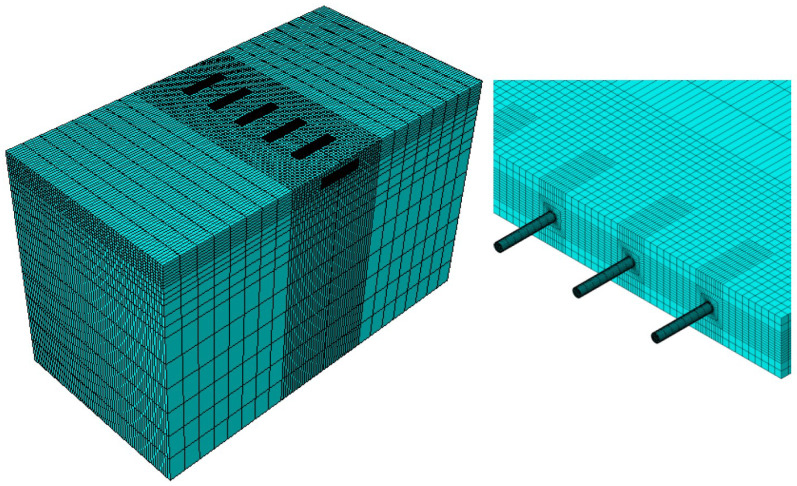
Mesh representation of the 3D model.

The interactions between the layers, the concrete slab, and the steel dowel bar were defined as surface-to-surface contact and modelled using Coulomb’s friction model, as illustrated in Figure 2a. A perfect bond was assumed between all of the layers, and therefore the coefficient of friction was set to 1.0. In contrast, the interface between the concrete slab and the dowel bar was modelled as a weak bond, with a coefficient of friction of 0.05. This weaker bond reflects the practical application of greasing the dowel bars before their installation in the concrete pavement to allow them to move in response to the thermal expansion and contraction of the concrete slabs. The boundary conditions have a significant role in the prediction of the model’s response. To accurately capture the behaviour of the JPCP, the boundary conditions were calibrated by comparing the model with the analytical solution. The bottom of the subgrade was fixed, while the edges of the pavement were constrained horizontally in the longitudinal (z-direction) and transverse (x-direction) directions, as well as vertically (the y-direction). The boundary conditions of the 3D model are shown in Figure 2b. Each part in the model was meshed using the linear-element C3D8Rs (eight-noded continuum elements). Adopting a smaller mesh size enhances the accuracy of the FE model results [57]. Therefore, a very fine mesh was utilised in the vicinity of the applied load, and to optimise the required computational time, a coarse mesh was employed in the region farther from the load, as shown in Figure 3.

## 4. Results and Discussion

### 4.1. Comparison of FE Analysis with Westergaard’s Central Loading Case

**Figure 4:**
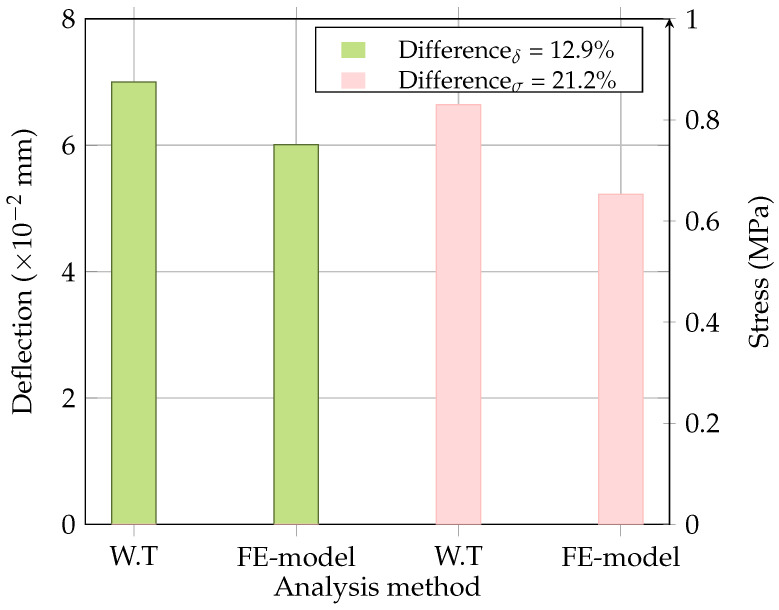
Comparison of deflection and stress values obtained from Westergaard’s theory (W.T) and FE model.

**Figure 5:**
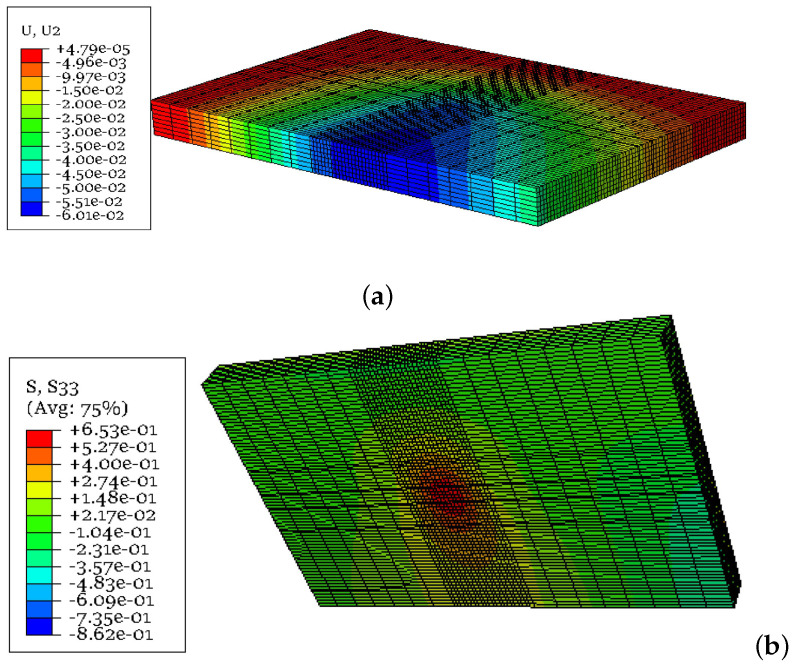
Stress and deflection distributions from FE model: (**a**) vertical deflection (mm) and (**b**) stress in the longitudinal direction (MPa).

The accuracy of the developed finite element model was checked using a well-known analytical solution, i.e., Westergaard’s theory for interior loading, where the concrete slab is loaded at the centre. The deflections (δ) and stresses (σ) obtained from the FE model and Westergaard’s solution are presented in Figure 4. It was observed that the deflection differed by around 13%, while the stress differed by approximately 21%, which was considered acceptable. This discrepancy is due to the model considering rectangular and finite boundaries; however, Westergaard’s method accounts for the circular loading and infinite boundaries. Figure 5 depicts the stress and deflection distribution obtained from the FE analysis.

### 4.2. The Effect of the Base Layer’s Stiffness on the Load Transfer and Flexural Stress at the Bottom of the Concrete Slab

**Figure 6:**
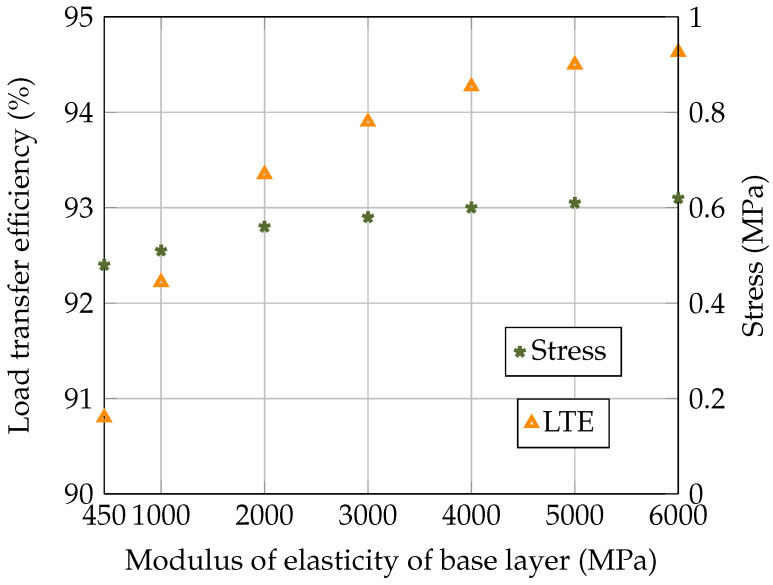
Effect of stiffness of the base layer on the load transfer efficiency and the flexural stress in the concrete slab.

After verifying the developed model, the dowel bar’s load transfer in the pavement system was analysed at various values for the stiffness of the base layer, i.e., 450 MPa, 1000 MPa, 2000 MPa, 3000 MPa, 4000 MPa, 5000 MPa, and 6000 MPa. The deflection values for the loaded and unloaded concrete slabs and the stress at the bottom of the loaded concrete slab are presented in Table 2, while the load transfer and the stress are plotted in Figure 6. It can be seen in Figure 6 that when the base layer was relatively flexible (with a low modulus of elasticity), the dowel bar significantly contributed to transferring the load across joints. As the modulus of elasticity of the base layer increased, the base carried more of the load, resulting in more consistent load transfer (with a smaller difference in the LTE) and reduced dependence on the dowel bars. Figure 6 also illustrated the relationship between the modulus of elasticity of the base layer and the flexural stress at the bottom of the concrete slab. As the base layer’s stiffness increased, the stress at the bottom of the concrete also rose; however, this change was not drastic. This is because the stiffer base supports the concrete slab, which generates less deformation and a marginally higher tensile stress. The analysis highlighted the importance of considering the base layer’s stiffness and the load transfer of the dowel bar in optimising the design of the concrete pavement to ensure its durability and long-term performance.

### 4.3. The Effect of the Base Layer’s Thickness on the Load Transfer and Flexural Stress at the Bottom of the Concrete Slab

**Figure 7:**
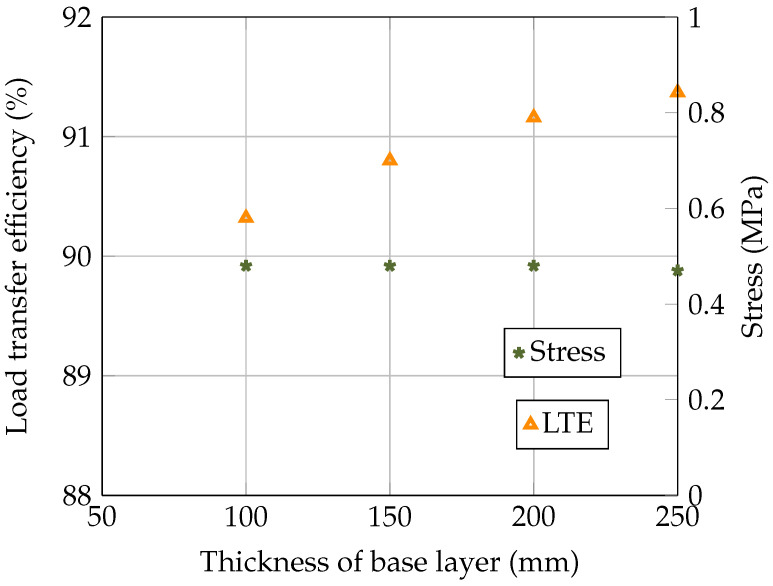
Effect of base layer thickness on load transfer efficiency and flexural stress in the concrete slab.

In this section, the structural response of concrete pavement is evaluated for different base layer thicknesses, i.e., 100 mm, 150 mm, 200 mm, and 250 mm. Table 3 provides the deflection values of loaded and unloaded concrete slabs and the stress at the bottom of the loaded concrete slab. The relationship between the thickness of the base layer, the LTE, and the stresses in the concrete slab is presented in Figure 7. It can be seen in Figure 7 that an increase in the base layer’s thickness led to an improved LTE. Furthermore, the flexural stress at the bottom of the concrete slab was observed to be 0.48 MPa for base layer thicknesses of 100 mm, 150 mm, and 200 mm. However, the stress marginally dropped to 0.47 MPa at a 250 mm thickness, indicating that the base’s thickness primarily supported the load transfer without affecting the internal stress behaviour of the concrete. For a practical design, selecting a base thickness that provides an adequate load transfer without excessive thickness can lead to a more economical and efficient pavement structure.

### 4.4. The Effect of the Dowel Bar’s Diameter on the Load Transfer and the Flexural Stress at the Bottom of the Concrete Slab

**Figure 8:**
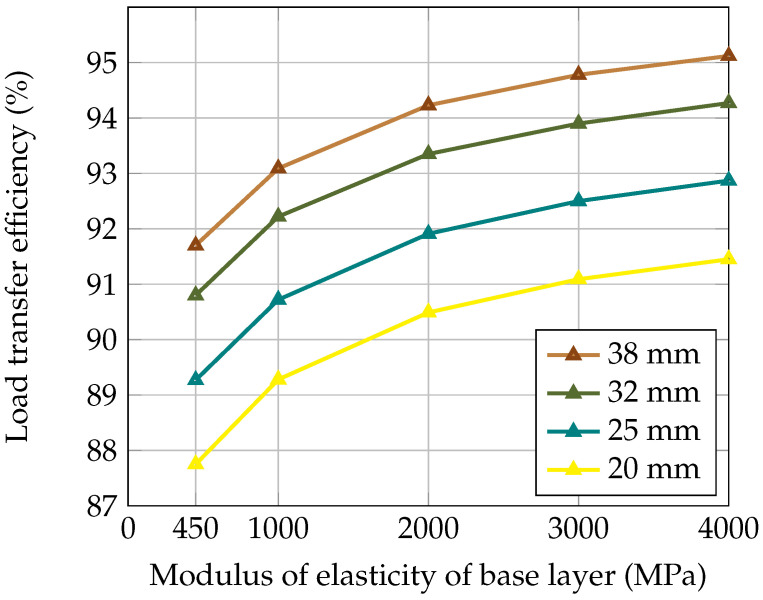
Effect of dowel bar’s diameter on load transfer efficiency.

**Figure 9:**
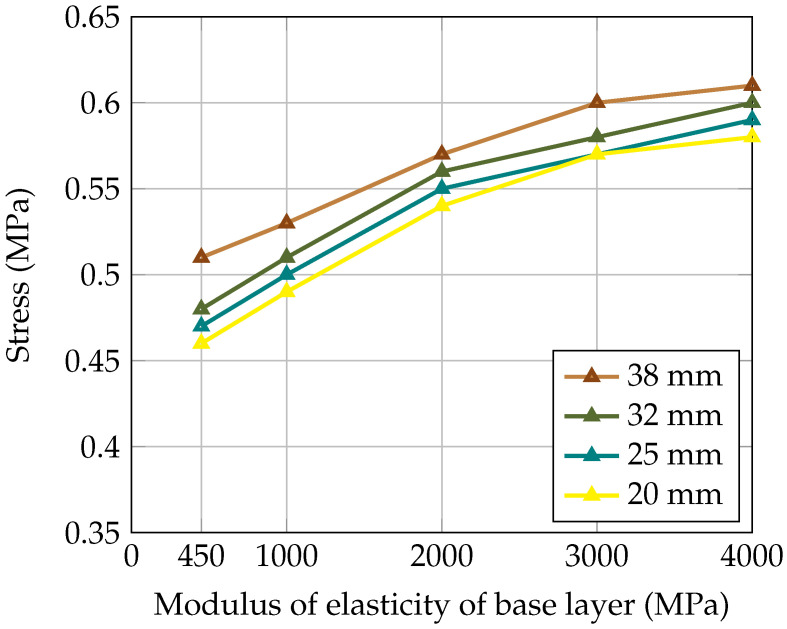
Effect of dowel bar’s diameter on flexural stress in the concrete slab.

In this section, the impact of the dowel bar’s diameter on the load transfer is evaluated based on there being different moduli of elasticity of the base layer. Table 4 displays the deflection values for loaded and unloaded concrete slabs and the flexural stress at the bottom of the loaded concrete slab. Figure 8 and Figure 9 illustrate the LTE and stress in the concrete slab for dowel bars with different diameters (20 mm, 25 mm, 32 mm, and 38 mm). It can be seen in Figure 8 that by increasing the dowel bar’s diameter, the LTE was improved, while the difference in the LTE was more pronounced for smaller diameters than for larger diameters. Moreover, Figure 9 shows that the smaller diameters resulted in lower stresses and larger diameters led to higher stresses. This is because the small diameters are more flexible, and a slight increase in size significantly improves their performance. In addition, flexibility reduces the stress concentration and thus mitigates potential cracking. In contrast, a larger diameter offers high rigidity and transfers the load over a smaller surface, thus increasing the possibility of localised damage to the concrete.

### 4.5. The Effect of the Dowel Bar’s Shape on the Load Transfer and Flexural Stress at the Bottom of the Concrete Slab

**Figure 10:**
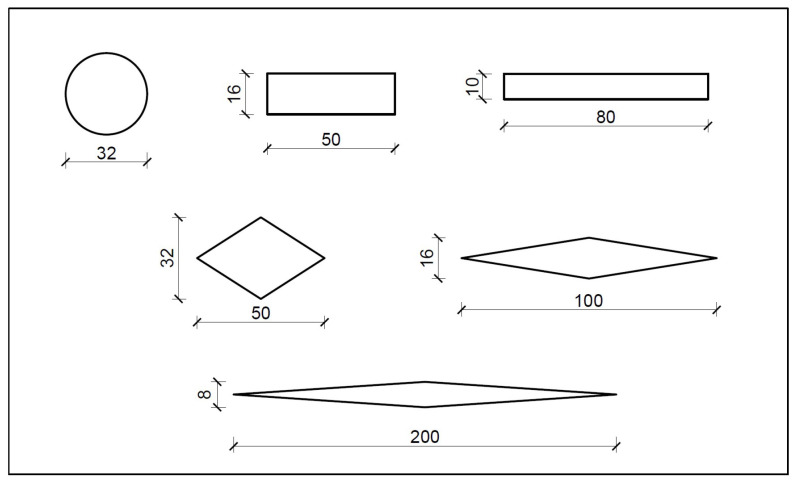
Various sizes of dowel bars [all dimensions are in mm].

**Figure 11:**
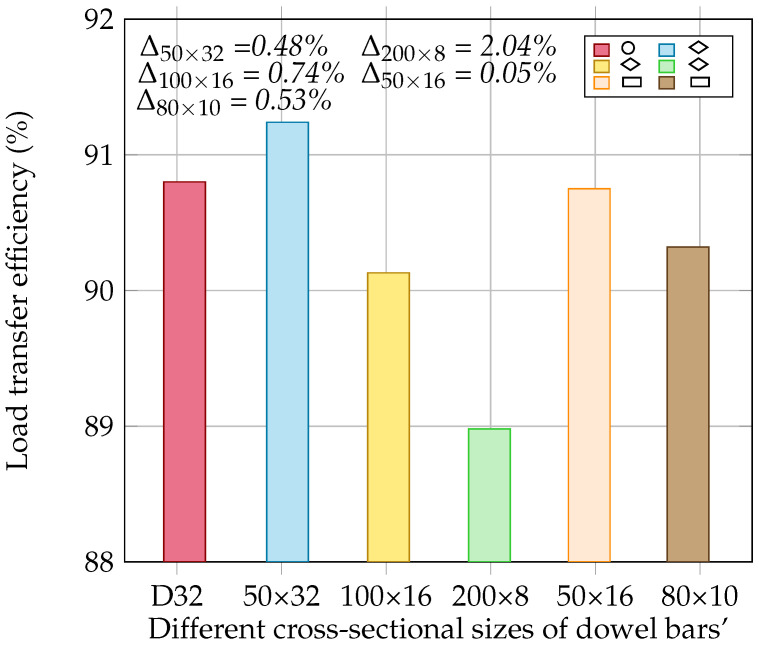
Effect of different dowel bar shapes on load transfer efficiency [the difference (Δ) in the load transfer efficiencies is calculated relative to that of D32].

In this section, the LTE of a steel rounded dowel bar with a diameter of 32 mm (D32) and the flexural stress at the bottom of the concrete slab are compared for various dowel bar configurations (diamond- and rectangular-shaped dowel bars) of different cross-sectional dimensions, i.e., 50 × 32 mm, 100 × 16 mm, 200 × 8 mm, 50 × 16 mm, and 80 × 10 mm; see Figure 10. The deflection values of the loaded and unloaded concrete slabs and the stress at the bottom of the loaded concrete slab are presented in Table 5. The load transfer efficiency of the rounded, rectangular and diamond-shaped dowel bars is presented in Figure 11. The results indicated that the diamond-shaped dowel bar of a 50 × 32 mm size substantially improved the load transfer compared to the rounded dowel bar, whereas the rectangular dowel bar of a 50 × 16 mm size provided a load transfer similar to that of the rounded dowels. However, the LTE decreased for diamond-shaped dowel bars with dimensions of 100 × 16 mm and 200 × 8 mm and for a rectangular dowel bar with dimensions of 80 × 10 mm. The higher load transfer in the diamond-shaped dowel bar of a 50 × 32 mm size indicates that this shape is more effective at distributing loads than the conventional rounded dowel bar. Conversely, the elongated dowel bars of 100 × 16 mm, 200 × 8 mm, and 80 × 10 mm sizes reduce the load transfer, indicating that overly elongated shapes are ineffective.

**Figure 12:**
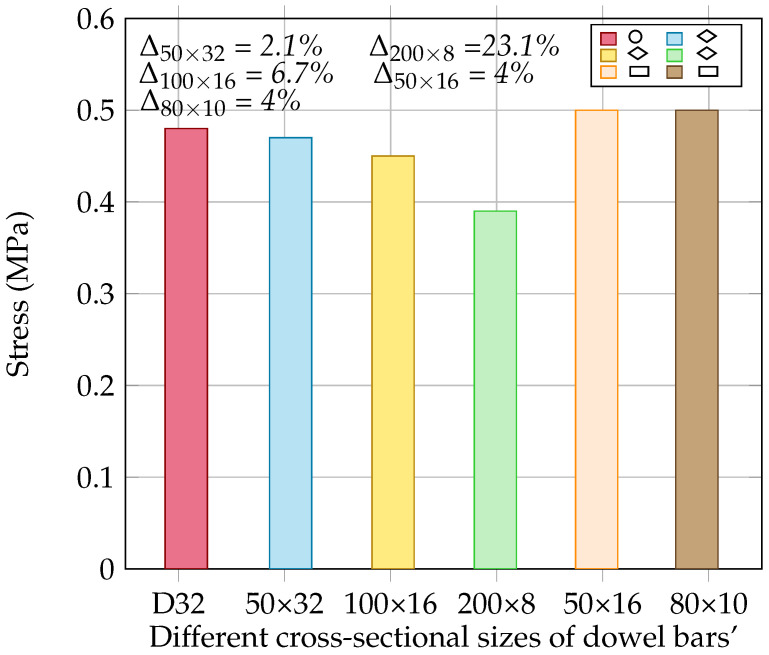
Effect of different dowel bar shapes on stress [the difference (Δ) in stresses is calculated relative to that with D32].

The impact of the different shapes and sizes of the dowel bars on the stress at the bottom of the concrete slabs is presented in Figure 12. It was observed that the dowel’s shape and size significantly influenced the stress distribution in the concrete slab. The diamond-shaped dowel bar with a size of 50 × 32 mm exhibited a slight reduction in stress compared to that with the rounded dowel, whereas the stress reduction was more pronounced for the sizes of 100 × 16 mm and 200 × 8 mm, indicating that an increase in the size of the diamond-shaped dowel bar reduced the stress in the slab. This is because the larger contact area of the dowel bar improves the interaction with the surrounding concrete, leading to a more uniform stress distribution in the concrete slab. However, rectangular dowel bars tend to induce a slightly higher stress in the slab than that with the rounded dowel bar. This increase in stress could be attributed to the geometry of the rectangular dowel bar, which creates sharper edges and causes a localised stress concentration that could contribute to the initiation of cracks, particularly under heavy traffic. On the other hand, the rounded dowel bar has geometric uniformity in its shape, and the diamond-shaped dowel bar features bevelled edges, which might help reduce the local stress concentration or crack formation by distributing the force over a large area. More importantly, the diamond-shaped dowel bar allows the concrete slab to move laterally in two directions. Therefore, diamond-shaped dowel bars are suitable for industrial or airfield pavements, where the concrete slabs are typically wider and undergo expansion and contraction in both directions while experiencing heavy loads. However, their adoption would depend on several factors, such as the design specifications, local construction practices, and economic considerations.

### 4.6. The Effect of Dowel Bar Length on the Load Transfer Efficiency and Flexural Stress at the Bottom of the Concrete Slab

**Figure 13:**
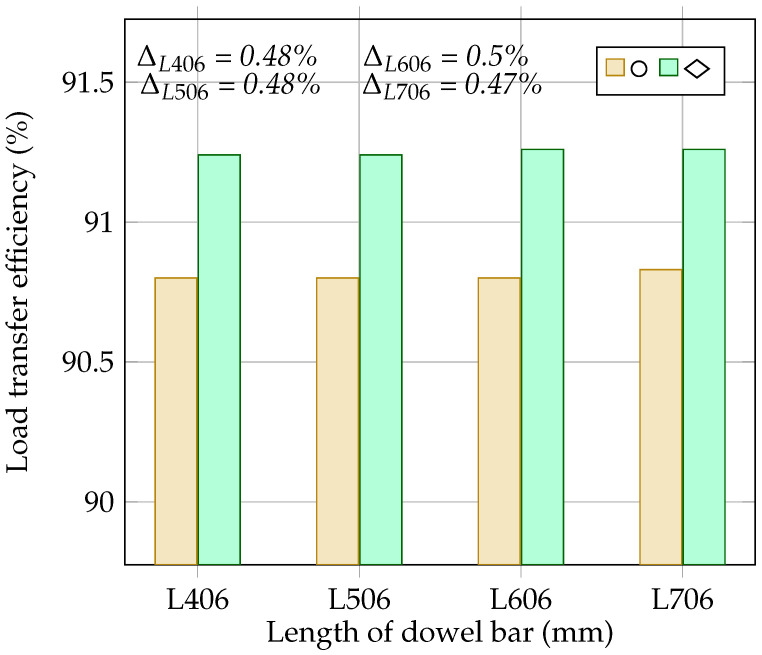
Effect of dowel bar length on load transfer efficiency.

**Figure 14:**
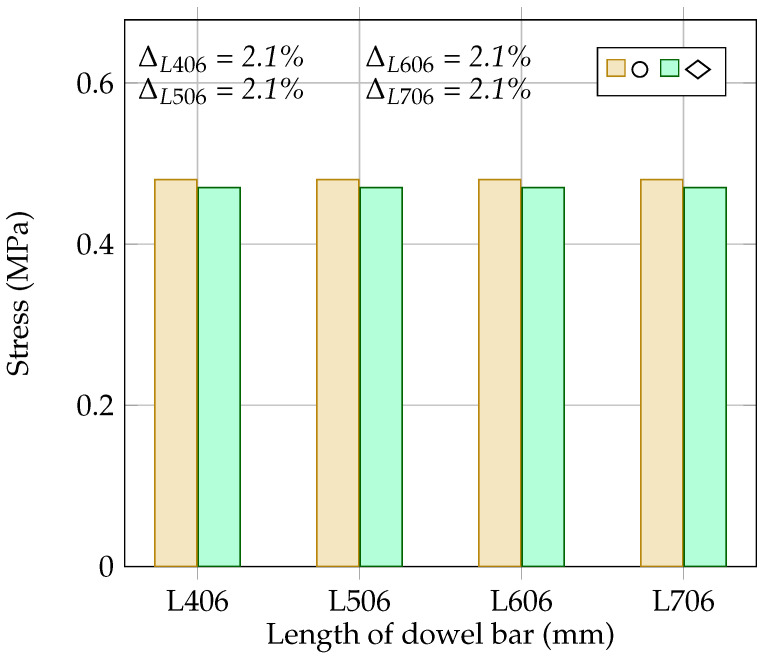
Effect of dowel bar length on flexural stress in the concrete slab.

This section discusses the impact of different dowel lengths [406 mm (L406), 506 mm (L506), 606 mm (L606), and 706 mm (L706)] on the load transfer across the joints and the stress at the bottom of the concrete slab for both the rounded dowel (D32) and the diamond-shaped dowel bar with a size of 32 × 50 mm. Table 6 represents the deflection values of the loaded and unloaded concrete slabs and the stress at the bottom of the loaded concrete slab. Figure 13 and Figure 14 show the relationship between the dowel bar’s length and the load transfer efficiency and the dowel bar’s length and the stress at the bottom of the slab, respectively. As shown in Figure 13, the LTE for the rounded dowel bar remained consistent for different dowel lengths, except for with the 706 mm dowel bar, where a noticeable increase was observed. For the diamond-shaped dowel bar, a slight increase in the LTE was observed for dowel lengths of 606 and 706 mm, while the dowel bar of a 506 mm length showed a similar LTE to that of the 406 mm long dowel. Figure 14 shows that the stress at the bottom of the concrete slab is consistent for both the rounded and diamond-shaped dowel bars across all lengths. Therefore, the selection of the dowel’s length should be tailored to the specific demands of the pavement, balancing the material cost, construction feasibility, and desired joint performance.

## 5. Conclusions

The following are the main conclusions based on the results of this study:

- With an increase in the modulus of elasticity of the base layer from 450 MPa to 6000 MPa, this study shows a 4% increase in the LTE and a 23% increase in stress. However, beyond 3000 MPa, the difference in the LTE and stress become more gradual, reaching approximately 0.38% and 3.2%, respectively.
- The results demonstrate that as the base layer’s thickness increases from 100 mm to 250 mm, the LTE improves by 1.2%. Conversely, the stress remains the same for thicknesses of 100 mm, 150 mm, and 200 mm, while a 2.1% decrease in stress is noticed at a thickness of 250 mm.
- The results show that increasing the dowel bar’s diameter significantly improves the LTE but also increases the stress in the concrete slab. However, the differences in the LTE and stress are more notable for smaller diameters and relatively flexible base layers. For base layer moduli of 450 MPa and 4000 MPa, the increase in the LTE and stress from a 20 mm to a 38 mm dowel bar were 4.3% and 3.8% and 10% and 5%, respectively.
- With a diamond-shaped dowel bar of a 50 × 32 mm size, a 0.48% increase in the LTE and a 2.1% decrease in stress are observed compared to these values for the rounded dowel bar, whereas diamond-shaped dowel bars with sizes of 100 × 16 and 200 × 8 show 0.74% and 2.0% decreases in the LTE, respectively, along with significant stress reductions of 6.7% and 23.1%. For a rectangular dowel bar with a size of 50 × 16, the LTE is approximately similar to that for the rounded dowel bar, while for a size of 80 × 10 mm, a 0.53% reduction in the LTE is noted. However, a 4% increase in stress is seen for both rectangular sizes.
- The diamond-shaped dowel bar improves the LTE by 0.48% for lengths of 406 mm and 506 mm and by 0.5% and 0.47% for lengths of 606 mm and 706 mm, respectively, compared to that with the rounded dowel bar. Additionally, the diamond-shaped dowel bar reduces the stress in the concrete slab by 2.1% relative to that with the rounded dowel.

## Figures and Tables

**Table 1:** Values of different parameters [56].

Materials	Modulus of Elasticity	Poisson’s Ratio
**-**	**MPa**	**-**
Concrete	33,000	0.2
Unbound gravel (base)	450	0.35
Gravel (subbase)	150	0.35
Sand (subgrade)	100	0.35
Steel dowel bar	200,000	0.35

**Table 2:** Deflection and stress values computed based on the stiffness of the base layer.

Elastic Modulus	Deflection at the	Deflection at the	Stress in
**of the Base Layer**	**Loaded Slab ($\delta_{L}$)**	**Unloaded Slab ($\delta_{u_{L}}$)**	**the Concrete Slab**
**MPa**	**mm**	**mm**	**MPa**
450	0.3860	0.3505	0.48
1000	0.3742	0.3451	0.51
2000	0.3624	0.3383	0.56
3000	0.3542	0.3326	0.58
4000	0.3475	0.3276	0.60
5000	0.3418	0.3230	0.61
6000	0.3368	0.3187	0.62

**Table 3:** Deflection and stress values computed based on the base layer’s thickness.

Thickness	Deflection at the	Deflection at the	Stress in
**of the Base Layer**	**Loaded Slab ($\delta_{L}$)**	**Unloaded Slab ($\delta_{u_{L}}$)**	**the Concrete Slab**
**mm**	**mm**	**mm**	**MPa**
100	0.3914	0.3535	0.48
150	0.3860	0.3505	0.48
200	0.3799	0.3463	0.48
250	0.3730	0.3408	0.47

**Table 4:** Deflection and stress values computed based on the diameter of the dowel bar.

Diameter of the Dowel Bar	Elastic Modulus of the Base Layer	Deflection at the Loaded Slab $(\delta_{L}$)	Deflection at the Unloaded Slab $\left(\delta_{u_{L}}\right)$	Stress in the Concrete Slab
mm	MPa	mm	mm	MPa
20	450	0.3927	0.3446	0.46
	1000	0.3796	0.3389	0.49
	2000	0.3668	0.3319	0.54
	3000	0.3580	0.3261	0.57
	4000	0.3510	0.3210	0.58
25	450	0.3896	0.3478	0.47
	1000	0.3772	0.3422	0.50
	2000	0.3648	0.3353	0.55
	3000	0.3562	0.3295	0.57
	4000	0.3494	0.3245	0.59
32	450	0.3860	0.3505	0.48
	1000	0.3742	0.3451	0.51
	2000	0.3624	0.3383	0.56
	3000	0.3542	0.3326	0.58
	4000	0.3475	0.3276	0.60
38	450	0.3833	0.3515	0.51
	1000	0.3720	0.3463	0.53
	2000	0.3605	0.3397	0.57
	3000	0.3525	0.3341	0.60
	4000	0.3460	0.3291	0.61

**Table 5:** Deflection and stress values computed based on the shape of the dowel bar.

Shape of the Dowel Bar	Cross-Sectional Size of the Dowel Bar		Deflection at the Loaded Slab $(\delta_{L}$)	Deflection at the Unloaded Slab $\left(\delta_{u_{L}}\right)$	Stress in the Concrete Slab
	Width	Height			
-	mm	mm	mm	mm	MPa
Rounded dowel bar (D32)	-	-	0.3860	0.3505	0.48
Diamond-shaped dowel bar	50	32	0.3857	0.3519	0.47
	100	16	0.3891	0.3507	0.45
	200	8	0.3938	0.3504	0.39
Rectangular dowel bar	50	16	0.3741	0.3395	0.5
	80	10	0.3702	0.3360	0.5

**Table 6:** Deflection and stress values computed based on the length of dowel bar.

Shape of the Dowel Bar	Length of the Dowel Bar	Deflection at the Loaded Slab $(\delta_{L}$)	Deflection at the Unloaded Slab $\left(\delta_{u_{L}}\right)$	Stress in the Concrete Slab
-	mm	mm	mm	MPa
Rounded dowel bar (D32)	406	0.3860	0.3505	0.48
	506	0.3860	0.3505	0.48
	606	0.3860	0.3505	0.48
	706	0.3873	0.3518	0.48
Diamond-shaped dowel bar	406	0.3857	0.3519	0.47
	506	0.3857	0.3519	0.47
	606	0.3857	0.3520	0.47
	706	0.3857	0.3520	0.47

## Data Availability

The original contributions presented in this study are included in the article. Further inquiries can be directed to the corresponding author.
